# Implementation and Application of an Intelligent Pterygium Diagnosis System Based on Deep Learning

**DOI:** 10.3389/fpsyg.2021.759229

**Published:** 2021-10-22

**Authors:** Wei Xu, Ling Jin, Peng-Zhi Zhu, Kai He, Wei-Hua Yang, Mao-Nian Wu

**Affiliations:** ^1^Department of Optometry, Jinling Institute of Technology, Nanjing, China; ^2^Nanjing Key Laboratory of Optometric Materials and Application Technology, Nanjing, China; ^3^Affiliated Eye Hospital of Nanjing Medical University, Nanjing, China; ^4^Guangdong Medical Devices Quality Surveillance and Test Institute, Guangzhou, China; ^5^School of Information Engineering, Huzhou University, Huzhou, China; ^6^Zhejiang Province Key Laboratory of Smart Management & Application of Modern Agricultural Resources, Huzhou, China

**Keywords:** intelligent diagnosis system, pterygium, anterior segment photograph, deep learning, diagnostic model training

## Abstract

**Objective:** This study aims to implement and investigate the application of a special intelligent diagnostic system based on deep learning in the diagnosis of pterygium using anterior segment photographs.

**Methods:** A total of 1,220 anterior segment photographs of normal eyes and pterygium patients were collected for training (using 750 images) and testing (using 470 images) to develop an intelligent pterygium diagnostic model. The images were classified into three categories by the experts and the intelligent pterygium diagnosis system: (i) the normal group, (ii) the observation group of pterygium, and (iii) the operation group of pterygium. The intelligent diagnostic results were compared with those of the expert diagnosis. Indicators including accuracy, sensitivity, specificity, kappa value, the area under the receiver operating characteristic curve (AUC), as well as 95% confidence interval (CI) and F1-score were evaluated.

**Results:** The accuracy rate of the intelligent diagnosis system on the 470 testing photographs was 94.68%; the diagnostic consistency was high; the kappa values of the three groups were all above 85%. Additionally, the AUC values approached 100% in group 1 and 95% in the other two groups. The best results generated from the proposed system for sensitivity, specificity, and F1-scores were 100, 99.64, and 99.74% in group 1; 90.06, 97.32, and 92.49% in group 2; and 92.73, 95.56, and 89.47% in group 3, respectively.

**Conclusion:** The intelligent pterygium diagnosis system based on deep learning can not only judge the presence of pterygium but also classify the severity of pterygium. This study is expected to provide a new screening tool for pterygium and benefit patients from areas lacking medical resources.

## Introduction

Pterygium is a common exterior ocular disease with unknown etiology. It is essentially a chronic conjunctival degeneration more common among people who live near the equator or work outdoors (e.g., fishermen and farmers) and is thought to be an irritative phenomenon due to ultraviolet light, drying, and windy environments (Coroneo, 2011; Delic et al., 2017). A pterygium is clinically divided into two phases: active and stationary. As a horizontal, triangular growth of the bulbar conjunctiva with the head extending toward the cornea, it appears as hypertrophy and hyperemia of the fibrovascular tissue in the active phase, with corneal infiltration. In the stationary phase, it shows no hyperemia, no or less fibrovascular proliferation, a flat head of the pterygium, and transparent cornea. If the pterygium in the active phase enlarges and encroaches on the pupillary area, it may cause vision loss with limited eye movement, pain, congestion, and other symptoms, as well as affect an individual’s appearance, and trigger astigmatism and higher-order aberrations (Lin and Stern, 1998; Gumus et al., 2012; Zhou et al., 2018). Currently, surgical resection is the main clinical treatment (Graue-Hernandez et al., 2019). If a patient is diagnosed early and treated with proper adjuvants, such as corticosteroids and mitomycin C, pterygium growth can effectively be controlled, and the recurrence rate could be reduced before and after surgical excision (Kaufman et al., 2013; Chen et al., 2015; Abdani et al., 2020). Early diagnosis helps to alleviate a patient’s pain, relieve their economic burden, and improve the quality of their vision.

Traditional screening methods for pterygium mainly depend on slit-lamp microscope observations and anterior segment photographs taken by ophthalmologists (Troutbeck and Hirst, 2001; Gumus et al., 2011). However, due to the lack of primary ophthalmologists, screening for pterygium still faces a huge gap in remote or rural areas with relatively limited medical resources. With the constant improvement of artificial intelligence (AI) theories and technologies, intelligent diagnosis and treatment have been rapidly growing in recent years; thus, many ophthalmologists and intelligent technologists have focused on relevant research. Deep learning was first used by the Google team to diagnose diabetic retinopathy (DR) through fundus images in 2016 (Gulshan et al., 2016). Since then different researchers have used deep learning models to detect DR (Kermany et al., 2018; Raman et al., 2018) and other fundus diseases, such as glaucoma (Li et al., 2018; Medeiros et al., 2020) and age-related macular degeneration (Nagasato et al., 2018; Yim et al., 2020). These studies obtained remarkable results and provided extensive ideas for clinical AI application. Some researchers have attempted using deep learning for ocular surface disease, the common one being pterygium. An iris segmentation method has been proposed to assess ptergium-infected tissues by analyzing anterior segment photographed images (ASPI) using digital image processing (DIP) algorithms (Abdani et al., 2015). Furthermore, a deep learning approach based on fully convolutional neural networks was set up for automatic detection and localization of the pterygium (Zulkifley et al., 2019). If we can utilize these techniques and develop a new intelligent method for consistent pterygium detection and mass screening, it will be beneficial for pterygium diagnoses and treatment. This study aims to investigate the application of a special intelligent diagnostic system, based on deep learning, in the diagnosis of pterygium. As a consequence, we expect that early detection and appropriate interventions could provide great convenience for ophthalmic patients.

## Materials and Methods

### Objects

The images used in this study were acquired from the Affiliated Eye Hospital of Nanjing Medical University. Anterior segment photographs were collected from a total of 1,220 patients (1,220 eyes) with/without pterygium from December 2019 to May 2021. Only one image was selected from each patient. For patients with pterygium, photographs of the worse eye were selected; for those with a normal ocular surface, photographs of a random eye were selected. The photos were selected by an experienced eye-surface specialist, and subsequent diagnoses were conducted by three ophthalmologists. The high-definition images selected in this study contained upper and lower lid margins, bulbar conjunctiva in the palpebral fissure area, and the whole cornea. The anterior segment photographs showed either normal appearance or a pterygium.

The exclusion criteria were as follows: (i) having conjunctivitis, subconjunctival hemorrhage, conjunctival cyst, conjunctival chemosis, conjunctival nevus, pseudopterygium, corneal conjunctival papilloma, and other ocular surface diseases; (ii) having had infectious keratitis, undergone corneal refractive surgery, and other medical histories; (iii) confusing signs like corneal scar or haze that affect the transparency of the cornea; and (iv) bad quality photographs, such as those out of focus or without appropriate light exposure.

This study was approved by the Institutional Research Ethics Committee of the Nanjing Medical University. All photographs were anonymized before inclusion in this study to ensure that they contained no information about the patients other than their diagnoses. These photographs were then randomly allocated into training samples (750 eyes) and the test set (470 eyes), and the training samples were divided into training and validation sets at a 9:1 ratio.

### Image Acquisition

All photographs were taken on a digital single-lens reflex camera (EOS 600D, Canon, Tokyo, Japan) that was integrated with a slit-lamp microscope as a digital slit-lamp image acquisition system (SLM-7E, Chongqing Kanghua Ruiming S&T Co., Ltd., Chongqing, China) using diffuse illumination, 10 × magnification, front view, an image resolution of 5,184 × 3,456, and an exposure time of 1/30 s. The operators were trained and qualified in a unified standardized anterior segment photographic technique.

**FIGURE 1:**
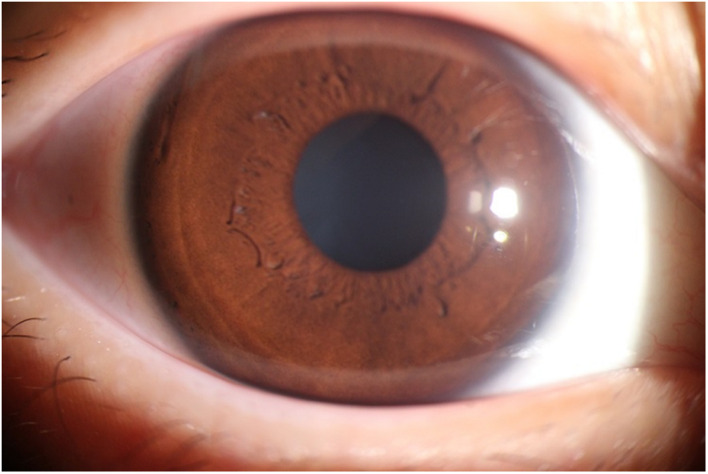
An example of the Normal group.

**FIGURE 2:**
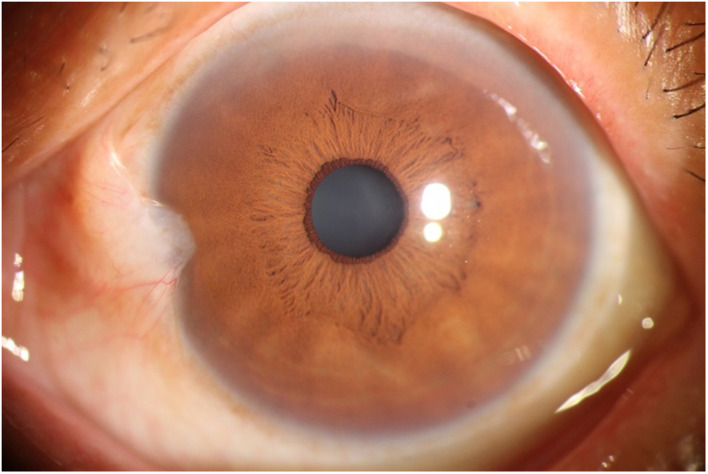
An example of the Observation group (pterygium).

**FIGURE 3:**
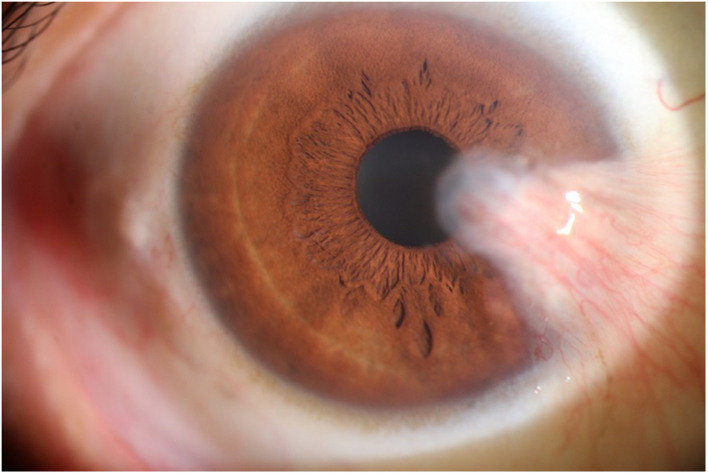
An example of the Operation group (pterygium).

The photographic images were classified into three categories (Lin and Stern, 1998) as follows: (i) normal anterior segment photographs with no obvious congestion or conjunctival proliferation, or corneal transparency (as shown in Figure 1); (ii) the observation group (pterygium) with the proliferative head extending beyond the corneal limbus <3 mm by horizontal length (as shown in Figure 2); and (iii) the operation group (pterygium) with the head extending beyond the limbus ≥3 mm by horizontal length (as shown in Figure 3).

### Model Training

This study was based on the deep learning software and hardware platform built by 20 Dawning graphics workstations with Graphic Processing Unit (GPU) M40, using a PyCharm integrated development environment to train a deep learning model. PyCharm is a powerful Python editor and a cross-platform that can improve the efficiency of program development. The 20 Dawning graphics workstations can satisfy the demand for a large amount of data training, which requires the support of a powerful GPU.

Based on EfficientNet-B6 (Tan and Le, 2019), we proposed a computer-aided diagnosis system for pterygium that relied on the anterior segment photography in this study. EfficientNets are a family of models obtained from a new baseline network designed by neural architecture, which uniformly scales all depth, width, and resolution dimensions using a compound coefficient (Tan and Le, 2019). It is an open-source project developed by Google with an Apache-2.0 license. EfficientNet-B0 acquires a better backbone with a neural network search (NAS) than previous algorithmic models. Being a variation from the family, EfficientNets-B6 is 1.8 times wider and 2.6 times deeper than B0, with the original image resolution reduced to 528 (Tan and Le, 2019).

Transfer learning is used to determine the initial parameters for systematic training without structural change in the EfficientNet model. We used parameters that have been trained on ImageNet as the initial parameters and then trained the model using photos collected and marked by our team to get a suitable model for this study.

A total of 750 anterior segment photographs previously diagnosed as normal or pterygium by the expert (250 eyes in each group) were used as training samples and randomly divided into training and validation sets at a ratio of 9:1 to train EfficientNet-B6. The classification criteria were based on the aforementioned methods.

**FIGURE 4:**
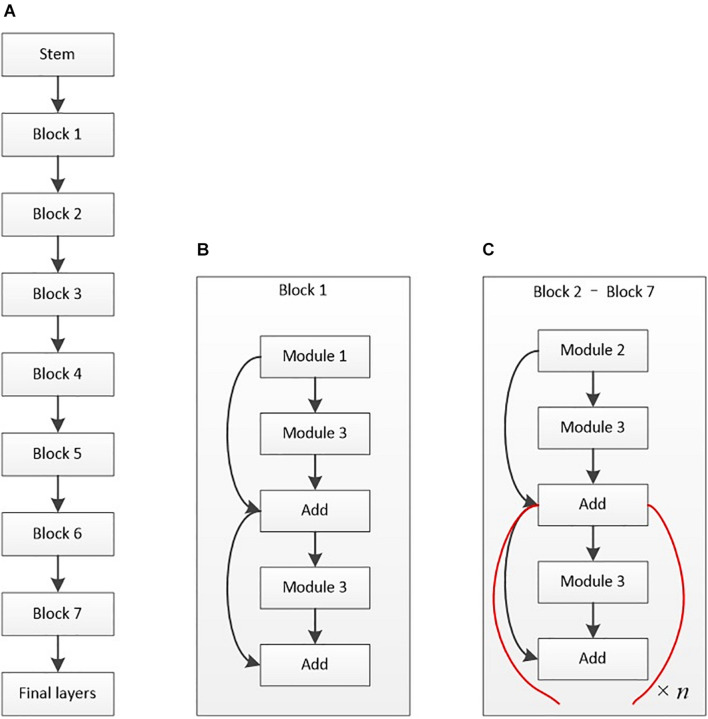
Architectural diagram of EfficientNet-B6. **(A)** Basic architecture. **(B)** Structure of block 1. **(C)** Structure of blocks 2–7. The basic structure of the stem is composed of a data input layer, convolution layer, activation function layer, and other layers. Module 1 mainly contains depth-wise convolution (D-Conv2D), batch normalization, and activation. Module 2 is primarily the connected two modules 1 with zero padding. Module 3 mainly contains global average pooling (GAP), rescaling, and Conv2D. The final layers include Conv2D, batch normalization, and activation layers.

With fewer parameters and efficient results, EfficientNet-B6 mainly includes a stem, seven blocks, and the final layers (as shown in Figure 4). The model’s batch size was 4, using the stochastic gradient descent method for optimization. A total of 50 epochs were trained, with the initial learning rate set at 0.01. Cosine annealing was used to reduce the learning rate during the training. On the basis of this framework, the parameters were adjusted during the training process.

The other 470 clear, canonical, and standard anterior segment photographs were then uploaded to the computer-aided diagnosis system, EfficientNet-B6, and judged by the artificial intelligence diagnosis technique. The results were obtained as the test set.

### Model Evaluation

An intelligent diagnosis was performed according to the photographic images of the anterior segment. The camera captured an anterior segment photograph of the interpalpebral zone, including the upper and lower lid margin and the whole cornea, at a right angle perpendicular to the iris, and uploaded it to the artificial intelligence diagnostic system. The photographs used in the study were in a uniform format without black edge or extra elements, and need no preprocessing. Before input to the model, the obtained photographs were resized to a slightly lower resolution for model and hardware reasons, and flipped horizontally at random during model training. When a photo was judged by the system, the report was obtained as the intelligent diagnosis group. The same photo will also be assessed by three eye-surface specialists independently in a double-blind trial on the same computer screen. The size of the pterygium was measured by the length of the slit light band of the slit lamp, and the grading diagnosis result of the pterygium was obtained according to the clinical diagnosis and treatment guidelines (Chinese Medical Association [CMA], 2007). Two or more identical grading diagnoses were used to create the final clinical diagnostic result. If two ophthalmologists provide unanimous grading diagnoses, that would be taken as the expert diagnosis; if two ophthalmologists provide different grades, a third ophthalmologist’s diagnosis would be considered and form the final result of expert diagnosis.

At present, there is no clear expert consensus on the indications for pterygium surgery; however, most ocular surface experts agree that the most important surgical indication is vision loss caused by the invasion of the visual axis (Twelker et al., 2000; Troutbeck and Hirst, 2001; Graue-Hernandez et al., 2019). In this study, a medium or large pterygium (horizontal length of the head extending beyond the corneal limbus ≥3 mm) warrants the recommendation for surgery (Wilson et al., 2008; Gumus et al., 2011). The sensitivity, specificity, kappa value, area under curve (AUC), and other indicators of the AI diagnostic system were calculated by comparing the results of intelligent diagnosis and expert diagnosis.

### Statistical Analysis

Statistical analyses were conducted with SPSS 22.0 (IBM Inc., Armonk, NY, United States), using methods for evaluating diagnostic tests, and represented by four grid tables. The enumeration data were represented by the number of images; indicators including accuracy, sensitivity and specificity, as well as F1-score and 95% confidence interval (CI) were expressed as percentages. Receiver operating characteristic (ROC) curves were plotted, and then the areas under the concentration-time curve (AUC) were calculated to measure the performance of the model. The kappa test was performed to evaluate the consistency of the diagnostic test. Taking the results of the expert diagnosis for the ground truth, a kappa value of 0.61–0.80 was considered significantly consistent, while a kappa value higher than 0.80 was considered highly consistent.

## Results

In this study, 470 anterior segment photographs were used to test the proposed intelligent diagnosis system for pterygium. According to the expert diagnosis, 189, 171, and 110 images were categorized into the normal, observation, and operation groups, respectively. The intelligent diagnostic system categorized 190, 162, and 118 images into these same groups respectively. The diagnostic results of the expert and intelligent diagnoses are listed in Table 1.

**TABLE 1:** Diagnostic results.

Expert diagnosis	Intelligent diagnosis			Total
	Normal	Observation (pterygium)	Operation (pterygium)	
Normal group	189	0	0	189
Observation group (pterygium)	1	154	16	171
Operation group (pterygium)	0	8	102	110
Total	190	162	118	470

**FIGURE 5:**
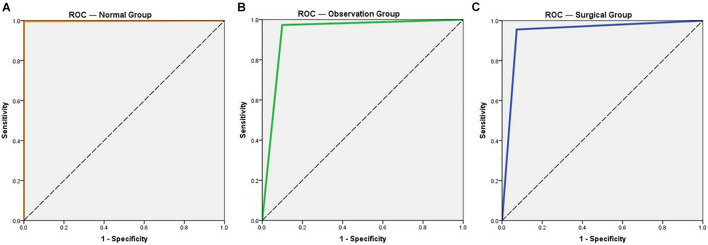
Receiver operating characteristic (ROC) curves of the three groups for normal eye and pterygium. **(A)** ROC curve of the normal group. **(B)** ROC curve of the observation group. **(C)** ROC curve of the operation group.

Compared with the expert diagnostic result, the true positive rate of the intelligent diagnostic system is almost 100% in the normal group. It also provides a high specificity of 99.64%. For both the observation and operation groups, the specificities of diagnosis for pterygium are above 95%, indicating the systems’ low misdiagnosis rates. The sensitivity for pterygium diagnosis is 90.06% in the observation group and 92.73% in the operation group, which is lower than that for the normal eye group. The kappa values for the normal group, the observation group, and the operation group is 0.996, 0.884, and 0.861, respectively. The AUC values of all three groups approaches 90%, with the highest value of 0.998 (for the normal group). Overall, the intelligent diagnostic system provides an average accuracy of 94.68% (Table 2). A comparison of the ROC curves of the normal, observation, and operation groups is shown in Figure 5.

**TABLE 2:** Evaluation index results.

	Evaluation indicators						
	Sensitivity	Specificity	F1-score	AUC	95% CI	Kappa	Accuracy
Normal group	100.00%	99.64%	99.74%	0.998	0.994–1	0.996	94.68%
Observation group (pterygium)	90.06%	97.32%	92.49%	0.937	0.909–0.965	0.884	
Operation group (pterygium)	92.73%	95.56%	89.47%	0.941	0.911–0.972	0.861	

*AUC, area under the curve; CI, confidence interval.*

## Discussion

Currently, there is no clear expert consensus regarding the surgical indications of pterygium. A survey conducted by Mexican investigators, covering 199 cornea specialists worldwide, shows that more than 90% of the specialists considered that surgery should be performed when the pterygium blocks up on the visual axis, or when there is pain, redness, eye movement restriction, or induction of astigmatism. In the same study, cosmesis was considered by 41.7% of the participants (Graue-Hernandez et al., 2019). For patients with pterygium in the stationary phase and without vision impairment, observations could be made temporarily without surgery. For pterygium in the active phase with vision loss, surgical resection should be performed. Although surgical treatments of pterygium vary, the indications for different operations are comparable (Troutbeck and Hirst, 2001; Janson and Sikder, 2014). Inappropriate surgical timing may cause unnecessary complications (Anduze and Burnett, 1996; Ti and Tan, 2003; Boui et al., 2020). The selection of medicines or surgical treatments relies on a doctor’s subjective judgment. Therefore, the development of an intelligent image processing method based on anterior segment photography, and the implementation of an AI-assisted automatic detection of pterygium, by which surgical indications could be identified through deep learning, will be beneficial for pterygium diagnoses. It not only ensures consistent pterygium detection but also enables mass screening for pterygium; therefore, the subsequent early detection and appropriate interventions will benefit patients.

At present, AI technology is being used more widely for anterior segment diseases, such as keratoconus, infectious keratitis, refractive surgery, corneal transplantation, cataract, angle closure glaucoma, dry eye and pterygium (Wu et al., 2020; Ting et al., 2021). A computer-aided pterygium screening platform developed by Zaki et al. has been used to classify pterygium and non-pterygium cases (Wan Zaki et al., 2018). This system is composed of four modules: the first uses the HSV color space and sigmoid transfer function to enhance the pterygium tissue in the image pre-processing; the second differentiates the pterygium tissue and the corneal region with a segmentation module; the third extracts corneal features with the circularity ratio, Haralick’s circulatory, eccentricity, and solidity; and the fourth identifies the presence or absence of pterygium by support vector machines and artificial neural networks. The sensitivity, specificity, and area under the curve of the pterygium screening system were 88.7, 88.3, and 95.6%, respectively. Recently, Zhang et al. have set up a deep learning diagnostic system to make diagnostic recommendations on whether a pterygium patient needs surgery, with a final accuracy of up to 95% (Zhang et al., 2018). Abdani et al. have utilized DeepLab V2 deep learning to increase the pterygium tissue segmentation performance from photographs taken on a mobile phone with an accuracy of 92% (Abdani et al., 2020). These results suggest that AI can classify pterygium based on its appearance.

In contrast to the above research, this study not only judged the presence of a pterygium using AI but also classified the observation and operation groups according to the horizontal length of the head extending beyond the corneal limbus in anterior segment photographs, therefore providing more accurate treatment recommendations. The results of the consistency analysis of the intelligent and expert diagnoses showed a high consistency (accuracy) of 445 eyes (94.68%). Among them, the sensitivity and specificity of the intelligent diagnosis system in the normal group were 100 and 99.64%, respectively. In the observation group, the sensitivity and specificity were 90.06 and 97.32%, respectively. Finally, in the operation group, the sensitivity and specificity were 92.73 and 95.56%, respectively. This study shows high consistency between intelligent and expert diagnoses when judging the presence of pterygium. However, the intelligent diagnosis is slightly less sensitive in grading the pterygium. This study shows high consistency between intelligent and expert diagnoses when judging the presence of pterygium. However, the intelligent diagnosis is slightly less sensitive in grading the pterygium.

Pterygium is one of the most common ocular surface disorders that causes vision loss and affects an individual’s appearance. It has a higher prevalence among people who live near the equator or work outdoors, being exposed to ultraviolet radiation, wind and dust. For the vast rural and remote areas where there is a lack of professional medical resources, especially ophthalmic specialists and products, intelligent diagnostic technology provides a convenient screening method for local patients with pterygium. It also aids communities by decreasing travel to distant county or city hospitals, reducing the economic burdens of the patients, and progressively providing treatment advice and clear further surgical indications. It will also be convenient for the timely referral of patients who need surgery in such situations as the global COVID-19 pandemic and will help facilitate the appropriate allocation of medical resources.

This study has some limitations. As the results suggested that the intelligent diagnosis was slightly less sensitive in grading the pterygium, further investigation and optimization of the diagnostic model are needed. All cases of mismatch in observation and operation groups were middle size pterygia, and the lengths of the proliferative head extending beyond the corneal limbus were between 2.5 and 3.5 mm, close to the critical value of 3 mm. For further research, the number of model training samples should be increased, and the acquisition of anterior segment photographs requires further optimization and screening because the quality of the images is very important for model training and testing. We only focused on the three-group classification of pterygium at this stage, there was no preprocessing of the original image before deep learning; We also continue working on localization of the lesions, involving segmentation and visualization for labeling and rating the image category.

Based on the relevant technical basis of this study, a new mobile terminal pterygium screening and diagnostic system will be developed in our future work, using photographs taken by a mobile phone as the training set. This system is expected to facilitate patients in remote or rural areas without anterior segment photographic facilities so that they can obtain a common eye disease diagnosis and treatment recommendations at home, with the aid of a smartphone.

## Conclusion

To meet the requirements of intelligent pterygium diagnosis, this study focused on breaking through the core theoretical models and key techniques needed for the initial screening diagnosis by the anterior segment photographs of pterygium. The intelligent pterygium diagnosis system based on deep learning can preferably classify pterygium. Intelligent diagnosis is highly consistent with expert diagnosis, especially in determining the presence or absence of pterygium. As the first study attempting to identify the severity of conjunctival proliferation, this study has made vital contributions by providing a new screening tool for pterygium to make basic diagnosis and treatment suggestions, and benefiting the majority of ordinary patients who lack medical resources. Future studies should aim to increase the number of training sets, constantly improve accuracy and sensitivity, and establish an intelligent pterygium diagnosis system suitable for basic use.

## Data Availability Statement

The raw data supporting the conclusions of this article will be made available by the authors, without undue reservation.

## Ethics Statement

This study was approved by the Institutional Research Ethics Committee of the Nanjing Medical University. Written informed consent from the patients/participants was not required to participate in this study in accordance with the national legislation and the institutional requirements.

## Author Contributions

W-HY and M-NW contributed to conception and design of the study and developed the intelligent system. WX, LJ, and P-ZZ organized the database. KH and M-NW conducted model training and algorithm adjustment. WX and LJ performed the statistical analysis and wrote the first draft of the manuscript. WX, LJ, P-ZZ, W-HY, and M-NW wrote sections of the manuscript. All authors contributed to manuscript revision, read, and approved the submitted version.

## Conflict of Interest

The authors declare that the research was conducted in the absence of any commercial or financial relationships that could be construed as a potential conflict of interest.

## Publisher’s Note

All claims expressed in this article are solely those of the authors and do not necessarily represent those of their affiliated organizations, or those of the publisher, the editors and the reviewers. Any product that may be evaluated in this article, or claim that may be made by its manufacturer, is not guaranteed or endorsed by the publisher.
